# Fabrication of Quasi-Sinusoidal Surface Relief Optical Transmission Gratings in Pyrex and IOG Glasses by Implantation with Oxygen and Nitrogen Ion Microbeams of the 5–6 MeV Energy Range

**DOI:** 10.1021/acsomega.4c01695

**Published:** 2024-07-04

**Authors:** István Bányász, István Rajta, Vladimír Havránek, María Cinta Pujol, Gábor Bazsó, György Kármán, Gyula Nagy

**Affiliations:** † HUN-REN Wigner Research Centre for Physics, P.O. Box 49, H-1525 Budapest, Hungary; ‡ Institute of Physics and Electrical Engineering, University of Miskolc, 3515 Miskolc, Hungary; § HUN-REN ATOMKI, Institute for Nuclear Research, P.O. Box 51, H-4001 Debrecen, Hungary; ∥ Nuclear Physics Institute AV CR, 250 68 Řež near Prague, Czech Republic; ⊥ Departament Química Física i Inorgànica, Universitat Rovira i Virgili, Campus Sescelades, E-43007 Tarragona, Spain

## Abstract

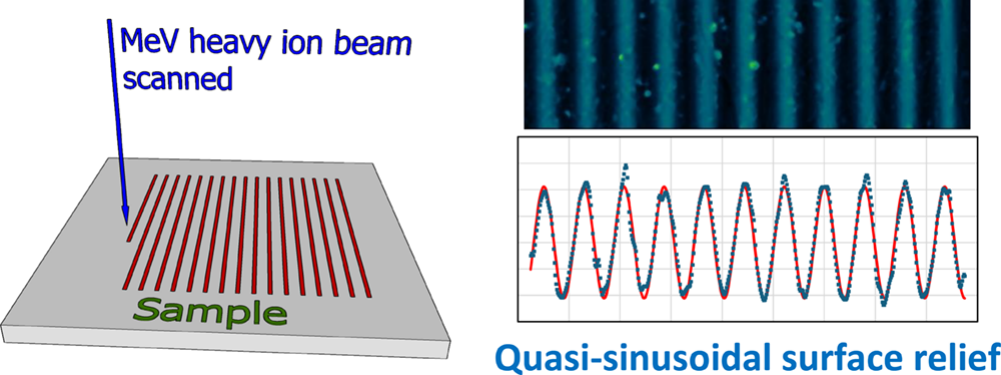

A method for the fabrication of high diffraction efficiency optical transmission gratings with quasi-sinusoidal profile in glasses by microbeams of medium-mass ions of 5–6 MeV energy was devised and demonstrated. Gratings with a 30 μm grating constant have been manufactured and characterized by interference microscopy and microprofilometry. The obtained surface profiles of the gratings were found to be quasi-sinusoidal with up to 265 nm amplitude. Measured highest first-order diffraction efficiencies were around 26% in both Pyrex and IOG glasses. Such gratings could serve as coupling elements in integrated optics and photonic integrated circuits.

## 1 Introduction. Previous Art

### 1.1 Basics of Diffraction Gratings

A diffraction or optical grating is an optical device that diffracts an incident light beam into various beams propagating at various directions that make various angles with the normal of the grating. In the case of white light illumination, the diffracted beams become colorful due to the structural colorization. Structural colorization is caused by the periodic structure of the grating of a period comparable to the wavelength of the light. In other words, diffraction gratings are dispersive.

Depending on the relative directions of the incident and diffracted beams, optical gratings are classified as reflection and transmission ones. Incident beams are reflected from a reflection grating as if it was a mirror. In the case of a transmission grating, the illuminating and diffracted beams propagate in the same direction.

In the greatest part of the diffraction gratings, there is a periodic modulation of either the index of refraction or the grating surface or both (mixed gratings).

Diffraction gratings also occur in nature too. Observation of a naturally occurring optical grating was first reported by Robert Hook. He described the wonderful colors of the peacock feather in his book Micrographia in 1665.^1^ James Gregory observed and described the behavior of a bird’s feather as a diffraction grating in 1673,^2^ just a year after Newton had discovered and published the dispersive properties of prisms.

It was David Rittenhouse who prepared the first known artificial diffraction grating in 1785. His diffraction grating consisted of 50 hairs extended between two fine thread screws. Line density of that grating was 4 line pairs per mm (lp/mm).^3^ Joseph von Fraunhofer, founder of optical spectroscopy and discoverer of the 574 dark lines in the emission spectrum of Sun, published the description of his diffraction grating in 1821.^4^

Thomas Young thoroughly studied diffraction orders and discovered the sinusoidal law of interference.^5^ Contributions of Augustin-Jean Fresnel in the field of diffraction gratings were also very important.^6^

The researchers who made the most important contributions to the development of the first diffraction gratings were Friedrich Adolph Nobert and William B. Rogers,^7^ as well as Henry Augustus Rowland.^8^

Excellent diffraction gratings were fabricated by Ányos Jedlik.^9^ His diffraction gratings of over 2000 lp/mm facilitated a subnanometer spectral resolution.

### 1.2 Modification of the Optical Properties of Materials by Ion Beam Implantation

Ion beam implantation has been used to modify the electric properties of materials since the 1960s.^10^

Implantation with high-energy ions, besides changing electric properties of the target, modify optical parameters of optical materials, such as glasses, crystals, and polymers in various ways. Implanted ions interact with the atoms of the target from their entry point at the sample surface down to the stopping range. Depending on the ion species and energy, either electronic interaction (with the electron shell of the target atoms) or the nuclear one (with the nucleus of the target atoms) get dominant. Basically, it is the index of refraction and absorption of the target that are changed. Surface relief changes due to ion beam implantation induced volume changes of the target can also be important. Townsend and his coauthors summarized optical effects of ion beam implantation in their monograph.^11^ There are some more recent monographs on the effects of ion beams on solids. Wesch and Wendler and their coauthors also deal in detail with all the optical effects caused by ion beam implantation or irradiation in solids.^12^ Chen et al. published a monograph on the use of ion irradiation for the fabrication of photonics structures in dielectrics.^13^ Besides the ion beam produced dielectric waveguides, they extensively deal with the synthesis of nanoparticles by ion implantation and the modification of the electrooptic properties, the photoluminescence, and the nonlinear optical properties of the dielectrics by ion beam implantation.

The first optical use of ion beam implantation was reported by Schineller and his coauthors.^14^ They fabricated optical planar waveguides in a SiO_2_ sample by proton beam implantation.

This field has evolved a lot during the last 55 years. Mainly planar and channel waveguides were fabricated by using various ion beam techniques. Homogenous macroscopic ion beams were used to fabricate the planar waveguides. Thin layers capable of guiding optical waves were obtained by this method. Ion beam implanted channel waveguides were fabricated either by using macrobeams with various masks or, more recently, by direct writing with ion microbeams. Parameters of ion beam implanted optical waveguides are comparable to or better than those obtained by other methods, such as ion exchange or laser writing. In many cases, ion beam implanted that optical waveguides can be used even at the “C” optical telecommunication band, around λ = 1500 nm. Chen published a thorough review article on this research field.^15^

Ion beam fabrication of periodic structures, i.e., transmission gratings in optical materials, also began rather early. In the first experiments on ion beam fabrication of optical transmission gratings, either macrobeam implantation through a mask or focused ion beam (FIB) implantation was used. Hartemann reported on fabrication of an acoustic surface wave resonator by implantation of a quartz substrate with a 100 keV He^+^ beam at a fluence of 1.5 × 10^16^ ion/cm^2^ through a mask.^16^ The spatial frequency of the implanted grating was 80 lp/mm, and its reflectivity at the Bragg resonance of 125 MHz was better than 99%. Garvin et al.^17^ used a holographically recorded photoresist mask and ion beam milling with an Ar^+^ ion beam of an energy of 3–10 keV to fabricate surface relief gratings on a GaAs sample. Grating constant was 370 nm.

Hwang and his coauthors fabricated ion beam implanted gratings in silicon solar cells.^18^ They implanted boron ions of the energy range of 20–180 keV at fluences between 10^11^ and 10^15^ ion/cm^2^. Then, by combining various fabrication steps, like chemical etching, with the implantation, they obtained Damman gratings with grating constants of 125 μm in both directions. A similar technique was used by Kurmer and Tang^19^ to produce grating couplers in optical waveguides in a LiNbO_3_ and a Corning glass sample, using B^+^ and N^+^ ions of energies of 200 and 170 keV at fluences around 10^16^ ion/cm^2^. The ion implanted grating coupler had diffraction efficiencies between 0.01 and 0.05%.

FIB devices also have been used in the fabrication of optical gratings. Erickson and his coworkers fabricated a phase mask for photo imprinting of fiber Bragg gratings. The phase mask was produced by FIB implanting a grating pattern into a fused silica sample using a 200 keV Si^2+^ beam of 100 nm diameter and subsequently wet etching the sample in a HF solution.^20^ Orth et al. reported on FIB fabrication of GaInAs/GaAs distributed feedback lasers in a series of articles.^21−23^ Yu et al. used FIB implantation with germanium ions to fabricate channel waveguides, grating couplers, Mach–Zehnder interferometers, ring resonators, and directional couplers in silicon.^24^

Direct writing of optical and other structures in materials using MeV energy ion microbeams began in the 1990s. Roberts and von Bibra reported on the fabrication of low-loss buried channel waveguides in fused silica by focused proton beam of an energy range of 1–3 MeV.^25^ Schrempel and Witthuhn proposed and developed a special technique for the production of three-dimensional microstructures, based on the use of 1.8 MeV proton beams of the width of 10 and 50 μm.^26^ Production of the microstructures involved selective etching of the proton beam irradiated regions of the sample.

Bettiol et al. published a review article on progress in proton beam writing in microphotonics.^27^ They reported on the successful application of MeV energy proton microbeam writing in the fabrication of channel waveguides, optical gratings, microlens arrays, and colloid crystal templates. They fabricated various optical gratings in poly(methyl methacrylate) (PMMA) samples. Proton microbeam implantation was followed by selective etching to complete the fabrication of the short-period gratings (600–1200 nm) of small lateral dimensions (100 μm × 30 μm).

Glass et al. used 3 MeV proton microbeams of 2–3 μm × 2–3 μm lateral dimensions and subsequent etching to produce various high aspect ratio surface relief structures in PMMA and SU-8 photoresist.^28^

Huszank et al. fabricated optical diffraction gratings and Fresnel zone plates in poly(dimethylsiloxane) (PDMS), using a 2 MeV focused proton beam of smaller than 2 μm diameter at high current of 200 pA. Lattice constants of the gratings were between 20 and 50 μm. No development was needed to obtain the final structures.^29^

More recently, Romanenko et al. reported on fabrication of gratings in PMMA polymer by using microbeams of 2 MeV protons^30^ and 10 MeV O^4+^ ions.^31^

In general, ion beam fabricated optical gratings had a low diffraction efficiency, and profiles of the individual grating lines were not easy to control.

Bányász et al. proposed and realized fabrication of high-spatial frequency ion beam implanted optical gratings in glass samples through photoresist masks, using light and medium-mass ions.^32^ They measured up to 18% first-order diffraction efficiency of the grating fabricated by 1.6 MeV N^+^ ions. Later, it was also proved that modulation of the optical path across the gratings was mainly due to the ion beam implantation induced surface relief (around 80%), and the rest was due to the changes in the refractive index (around 20%).^33^ In the case of the finest gratings with 4 μm grating constant, the surface relief profile was found to be quasi-sinusoidal. This fact was attributed to the lateral straggling of the implanted ions.^33^

It must be noted that the above review of previous research was focused on the modifications most relevant to the research presented in this review, where the target materials were glasses. As mentioned earlier, other properties, such as photoluminescence and optical nonlinearity of crystals, could also be modified by ion beam implantation. There are extensive researches in those fields too.^34^

### 1.3 Other Methods for the Fabrication of Microgratings and Other Integrated Optical Elements and Devices

Besides the ion beam techniques presented in Section 1.2, there are two main methods for the fabrication of integrated optical elements and devices. They are direct laser writing and the use of outdated microelectronic fabrication facilities (photonics foundries).

The results on direct writing of optical and other microstructures by focused laser beams in various targets date back to the late 1980s and early 1990s.^35^ The target materials for the first laser-written micro-optical elements were photoresists or other photosensitive materials. A development step was included, and replicas were fabricated by hot stamping with the metallized master element.

The invention and widespread use of femtosecond lasers allowed for the fabrication of micro-optical structures of higher resolution in various optical materials through several processes, like various forms of intensity-dependent nonlinear absorption.^36,37^ According to these reviews, common challenges of those fabrication methods were the spatial forming of the femtosecond laser pulses, such as the elliptical cross section of the beam and the aberrations arising when tightly focusing the beam into a transparent dielectric. Due to the extremely high intensity at the focal spot, Kerr self-focusing also occurs.

More recent achievements were reported in laser writing of micro-optical structures in optical crystals, especially in diamond.^38,39^ The above articles involved sophisticated fabrication schemes, including the use of a high-intensity femtosecond pulse, followed by a lower-intensity pulse train.

Geudens and coworkers have recently demonstrated the feasibility of the fabrication of femtosecond laser micromachined 3D glass photonics platform in fused silica substrate.^40^ Again, the fabrication process was intricate, involving femtosecond laser direct writing and femtosecond laser irradiation, followed by chemical etching.

Photonics foundries basically use outdated microelectronic fabrication facilities. While the current minimum feature size in the electronic integrated circuits is a few nanometers, the typical minimum feature size of photonic integrated circuits (PICs) is a few tens of nanometers. Such fabrication methods have been applied to various photonic substrates and platforms.

Van der Tol et al. published a review article on the comparison of monolithic techniques for the fabrication of InP-based photonic circuits in 2010.^41^ A more recent review on recent progress in InP PICs was published by Soares et al.^42^

Other types of PICs include silicon PICs,^43^ Si_3_N_4_ PICs,^44^ and others.

Since PICs consist of various materials and a number of building blocks, their fabrication is more complicated than that of the older electronic integrated circuits.

### 1.4 Aims of the Research

Thanks to the availability of modern Tandetron accelerators with microbeam facilities, we decided to perform extensive experiments to fabricate quasi-sinusoidal optical gratings by using high-energy (5–6 MeV) microbeams of medium-mass ions (nitrogen and oxygen) in optical glasses. As it can be seen in Section 1.2, previous work in writing of gratings and other optical structures using ion microbeams did not produce continuous optical path profiles (refractive index and surface relief) across the grating lines or the smallest elements of other optical structures. The novelty of the method proposed and demonstrated by us is that the fluence of the writing ion microbeam was changed from pixel to pixel to obtain the desired quasi-sinusoidal profile of the gratings. As it was mentioned at the end of Section 1.2, we based these researches on the results of our previous research projects on fabrication of optical elements by ion beam implantation.

## 2 Results and Discussion

### 2.1 Design of the Gratings

The aim of our research was to produce high-spatial frequency optical gratings of a quasi-sinusoidal profile with high diffraction efficiency in optical glasses using microbeams of medium-mass ions. The ultimate higher limit to the spatial frequency (and the lower limit to the grating constant) was determined by the lateral dimensions of the microbeam.

The advantage of sinusoidal optical gratings over gratings of any other profile is that besides the nondiffracted zeroth order, all the incoming light is diffracted into the first orders.

As explained in Chapter 1.2, according to our earlier results on the fabrication, the optical gratings by implantation through a mask, lateral straggling rendered the finest diffraction gratings of a grating constant of 4 μm quasi-sinusoidal.^33^ Since the lateral dimensions of the ion microbeams used in our actual experiments were in the range of 1.5–2.4 μm, we could expect quasi-sinusoidal grating profiles when writing “binary” gratings with the microbeams. In other words, writing single stripes separated by a grating constant of 3–5 μm would result in quasi-sinusoidal grating profiles. Amplitude of the surface relief grating would depend on the ratio of the grating constant to the ion beam width. Moreover, properties of the target material also strongly influence the achievable amplitude, e.g., the same implantation parameters would result in higher amplitude surface relief structures in polymers than in glasses or crystals. Sign of the surface change (i.e., swelling or compaction) also depends on the target material.

For the case when the ratio of the grating constant to the ion beam width is higher than 2, we have developed a method to fabricate gratings of a quasi-sinusoidal profile. This method relies on the assumption that, in an adequate range of implanted fluences, the swelling or shrinkage of the target material caused by the ion microbeam is proportional to the implanted fluence. Effects of swift heavy ion irradiation on the structural and volume changes of the target material can be treated in the framework of the thermal spike model. The thermal spike model was first proposed in the first half of the last century by Dessauer.^45^ The model was extended to swift heavy ions by Jordan.^46^ The modern form of the theory of thermal spikes was developed by F. Seitz and his coauthors.^47,48^ In our case, this effect is mainly local swelling of the target surface under the microbeam. Such volume changes can be explained by the phenomenon of plastic deformation of the target in the microregions of the ion tracks.^49−52^ Of course, the energetic ion microbeams induce changes under the target surface as well, changing the structure of the material and hence its optical properties too.

To achieve a quasi-sinusoidal profile of those ion microbeam implanted gratings, we wrote each period using multiple passes of the microbeam, and fluence was changed from pass to pass (keeping the beam parameters constant) so that the resulting distribution of the implanted fluence in the direction of the grating constant be sinusoidal.

**Figure 1:**
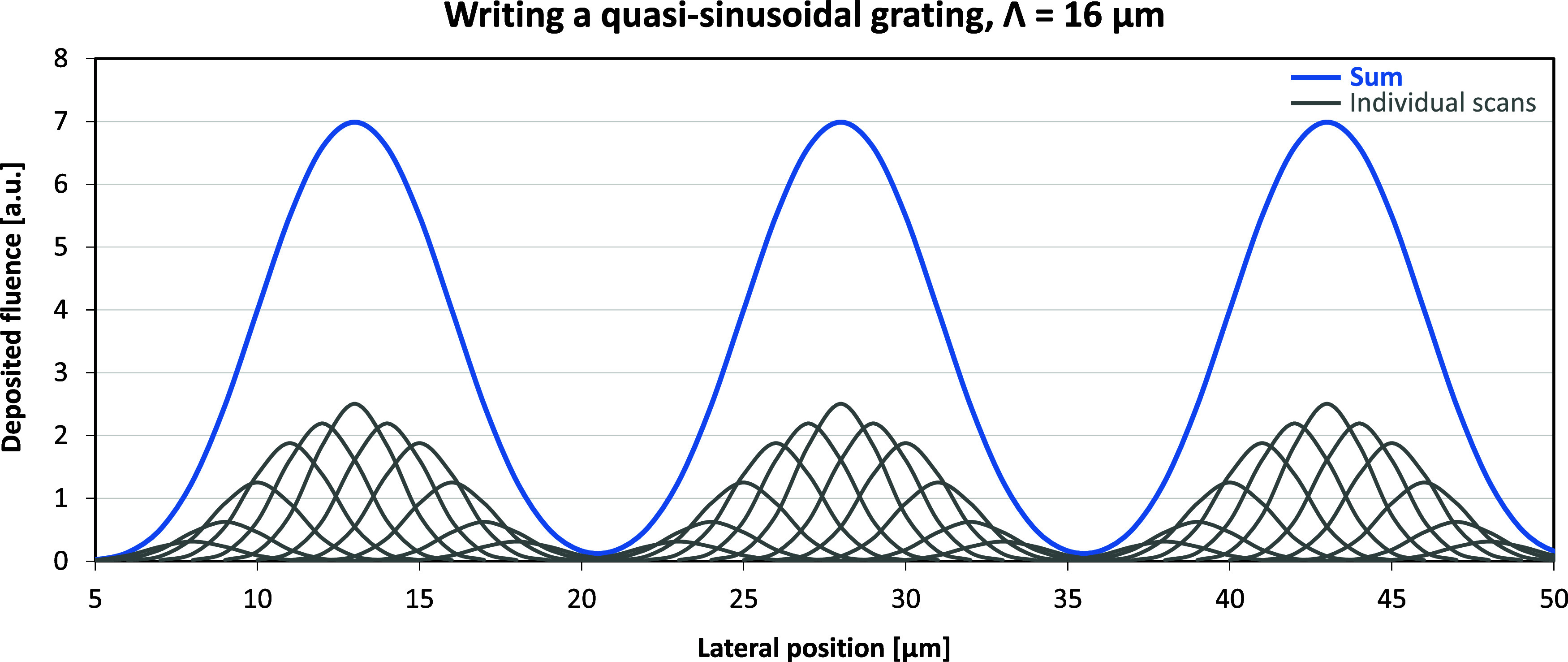
Distribution plan of the deposited fluence across the substrate to achieve quasi-sinusoidal gratings of 16 μm. Profile of the ion microbeam is Gaussian with a fwhm of 3 μm. Scans were made with a step size of 1 μm. Fluence distributions of the individual scans are represented by gray lines. Total deposited fluence distribution is represented by the blue line.

An example of grating planning is shown in Figure 1.

It can be seen that, if the height of the local surface swelling is linearly proportional to the locally deposited total fluence, a surface relief grating of quasi-sinusoidal profile could be obtained.

### 2.2 Implantation of the Gratings

We have designed and fabricated optical transmission gratings of sinusoidal profile in optical glasses using high-energy nitrogen and oxygen microbeams at the Tandetron laboratory of the UJF research institute in Řež, Czech Republic. The results presented here were obtained in Pyrex^53^ and IOG^54^ glasses. Conditions of the various implantation experiments are presented in Table 1.

**Table 1:** Implantation Parameters of the Studied Gratings and the Amplitude of the Sine Function Fitted to Their Surface Relief Profiles

grating group	sample material	implanted ion and charge	ion energy [MeV]	current density [10^–3^ A/cm^2^]	implanted fluence [ion/cm^2^]	Λ [μm]	amplitude of the fitted sine curve [nm]	error of the fitted amplitude [nm]
GG	IOG glass	N^3+^	5	1.73	8 × 10^14^	30	211	2
GF2	Pyrex glass	O^4+^	6	4.17	8.4 × 10^15^	30	265	5
GF4	Pyrex glass	N^3+^	5	1.07	8.4 × 10^15^	30	193	6

Sinusoidal modulation of the implanted fluence across the gratings was achieved in the following way. Each grating line was divided into stripes of equal width equal to the width of the microbeam. The desired sinusoidal distribution of the fluence was quantized, and each stripe was assigned the quantized fluence, normed to the maximum of the sine distribution. Then, the dwelling time for each pass over the stripes was calculated by dividing the fluence by the ion beam flux. The microbeam passed only once over each stripe.

### 2.3 SRIM Simulations

Predicting the effects of ion beam implantation on a target material is very difficult, since they depend strongly on the composition and structure of the target material, the species of the implanted ions, their energy, the ion beam current, and the implanted fluence.^11^ In the case of ion microbeams, the beam size is also an important factor, as current density is inversely proportional to the beam area and the effects of the implantation strongly depend on the current density in the ion beam.

Interaction of the implanted ions with the target material can be simulated by various computer codes. We used the Stopping and Range of Ions in Matter (SRIM) code^55^ to get a rough estimation of the possible effects of ion implantation in our experiments.^56^

**Figure 2:**
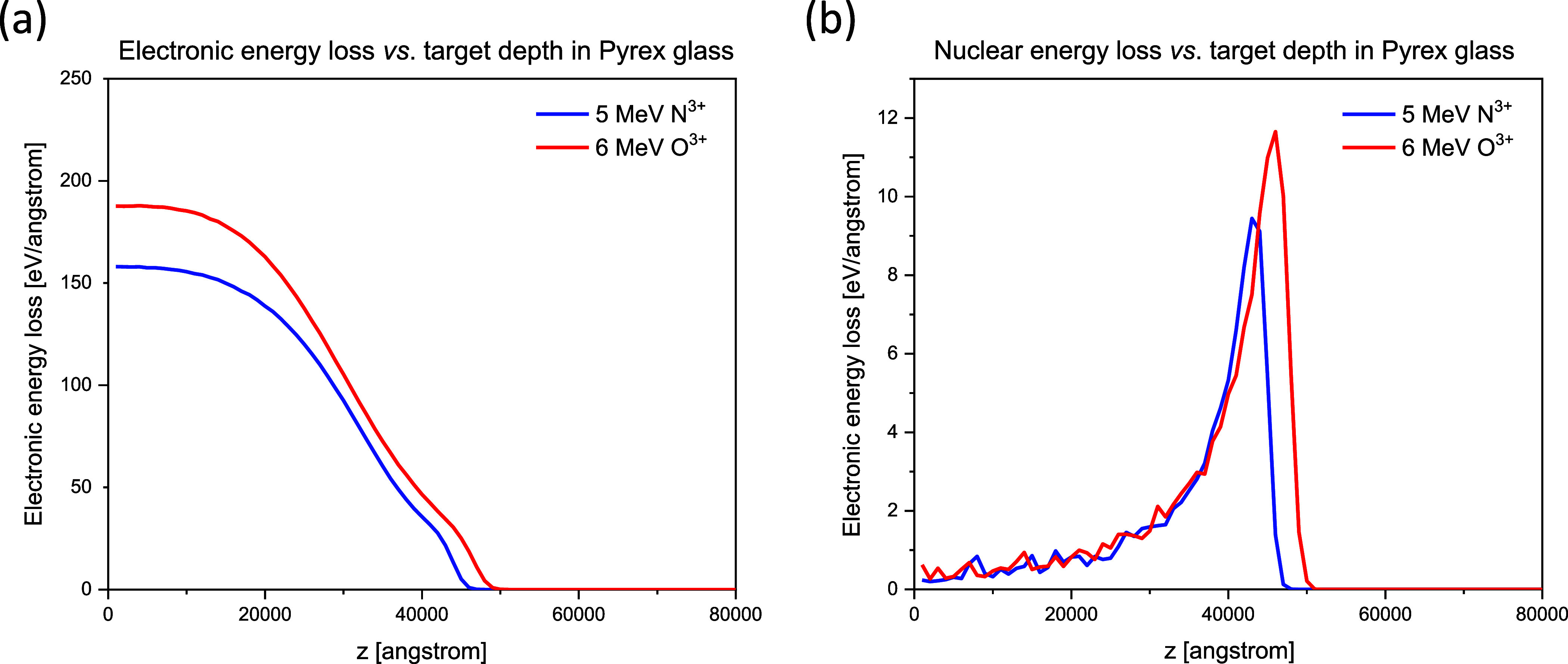
Electronic (left) and nuclear (right) energy loss vs. depth in Pyrex glass of 5 MeV N^3+^ and 6 MeV O^4+^.

**Figure 3:**
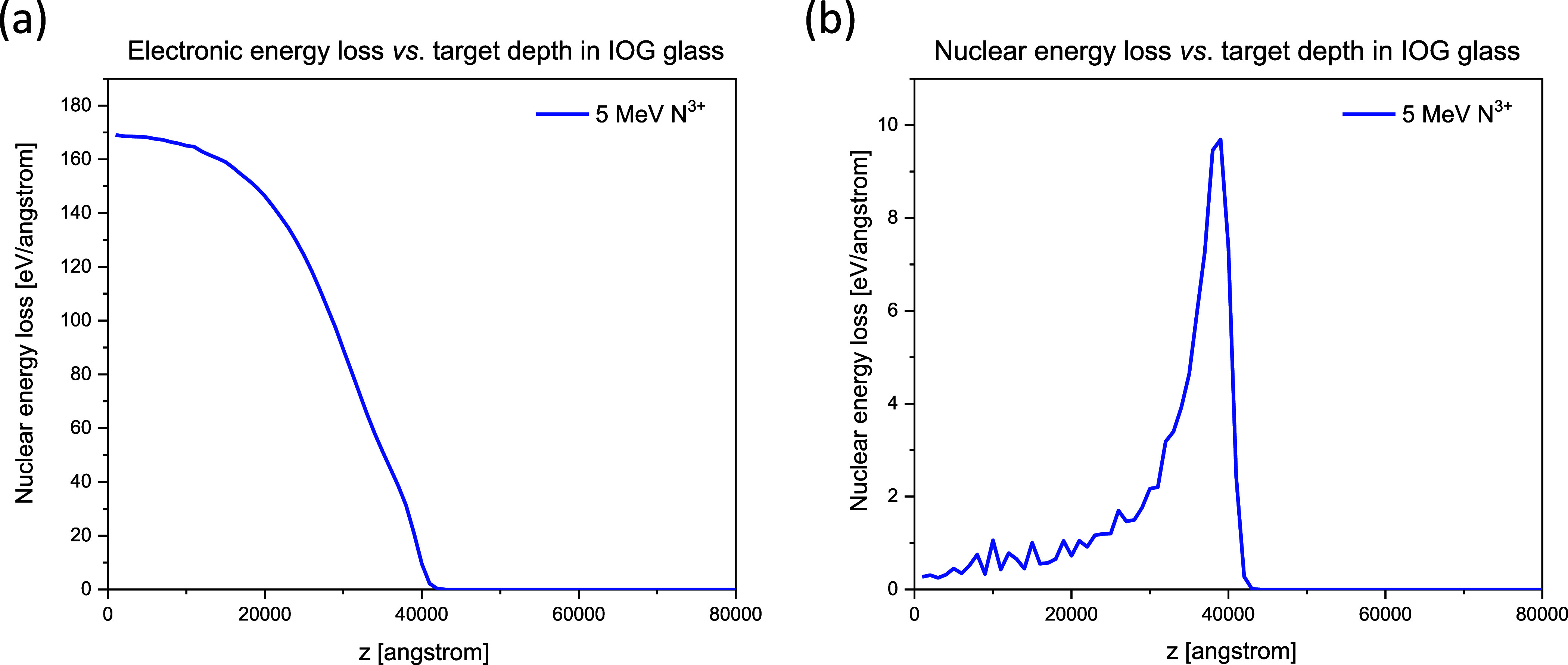
Electronic (left) and nuclear (right) energy loss vs. depth in IOG glass of 5 MeV N^3+^.

Both electronic and nuclear stopping powers vs target depth for all the combinations of implanted ions and target materials have been calculated. The results are listed in Figures 2 and 3.

Maximum of electronic energy loss was between 150 and 180 eV/angstrom for all the implanted ions at all the energies in both target materials.

Position of the sharp maximum of the nuclear energy loss (the Bragg peak) depends on the sample material composition, the ion species, and the ion energy. Height of the Bragg peak remained in the 9–12 eV/angstrom range in the studied ion–target combinations. Electronic energy loss was more than an order of magnitude higher than the nuclear one.

One can expect a higher amplitude of the ion beam induced swelling when the peak electronic energy loss is higher.

### 2.4 Microscopic Study of the Gratings

All of the ion microbeam implanted gratings were studied using a Zeiss Peraval transmission optical microscope in both interference and interference phase contrast (INTERPHAKO) modes.

**Figure 4:**
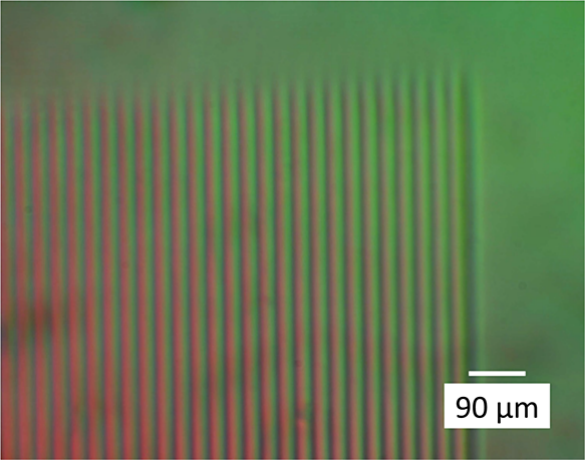
INTERPHAKO microphoto of the grating GG, implanted in IOG glass by 5 MeV N^3+^ ions. Grating constant is 30 μm. 6.3× objective, 16× illuminating grating.

**Figure 5:**
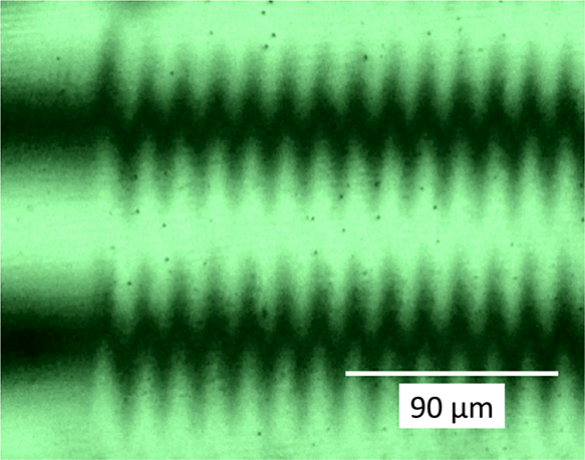
Interference microscopic microphoto of grating GG, implanted in IOG glass by 5 MeV N^3+^ ions. 12.5× objective, 63× illuminating grating, green interference filter centered at 541 nm.

The ion microbeam implanted gratings are basically phase objects. Variations in the optical path across the microscopic image of the phase objects are transformed into interference fringes in the interference microscopy mode, while they are transformed into various interference colors in the INTERPHAKO mode. Typical microscopic photographs of the ion microbeam implanted gratings are shown here in Figures 4 and 5.

As it was stated earlier, ion beam implantation results in mixed gratings, i.e., both the index of refraction and the surface height are modulated. The abovementioned two modes of transmission optical microscopy show the total modulation of the optical path across the sample, i.e., the algebraic sum of the above two kinds of modulation. The only way to determine separately the amplitudes of the refractive index and surface relief modulation could be the repetition of the microscopic measurements with an immersion-type objective using an immersion liquid with an index of refraction equal to that of the surface of the sample. Thus, surface relief modulation could be (at least partially) canceled.

### 2.5 Microprofilometric Study of the Gratings and Analysis of the Grating Profiles

**Figure 6:**
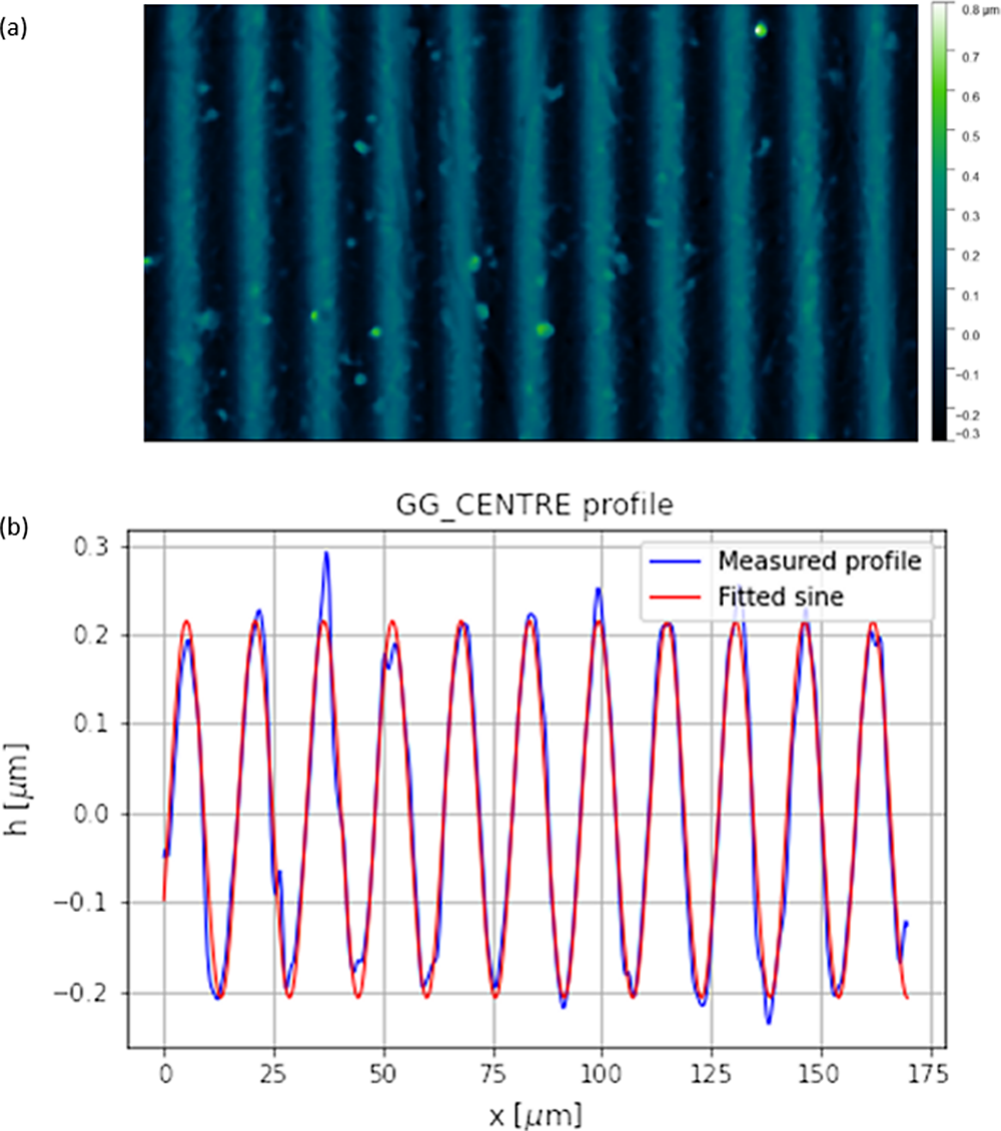
(a) Surface topography of grating GG implanted in IOG glass. (b) Measured profile (blue line) and fitted sine curve (red line) of grating GG implanted in IOG glass.

A high-resolution optical profilometer, Sensofar PLU 2300, was used for the systematic study of the ion microbeam implanted surface relief gratings.^56^ Grating profiles were extracted from the surface topographies, and then, sine functions were fitted to the profiles. Typical results are presented in Figure 6.

It can be seen that the ion microbeam implanted surface relief gratings were mainly of a regular, generally smooth profile.

The results of the profilometric studies of the surface relief gratings, as well as the conditions of the various implantation experiments, are summarized in Table 1.

The surface profiles of our fabricated gratings were fitted with sine curves, and the relative error of the fits was 3% at worst.

In the case of Pyrex glass targets, the highest amplitude surface relief grating, 265 nm, was obtained by implantation with O^4+^ ions of 6 MeV, at a peak fluence of 8.4 × 10^15^ ion/cm^2^. When 5 MeV N^3+^ ions were implanted at the same fluence into the same material, the amplitude of the surface relief grating was 193 nm. Significant differences in the amplitude obtained by nitrogen and oxygen ions at the same fluence and slightly different energies could be explained by the higher current density of the oxygen ion beam (4.17 × 10^–3^ A/cm^2^) compared to that of the nitrogen ion beam (1.07 × 10^–3^ A/cm^2^).

Implantation of a 5 MeV N^3+^ ion microbeam resulted in high (211 nm) amplitude surface relief gratings nm in IOG glass.

### 2.6 Measurement and Calculation of the Diffraction Efficiencies

**Figure 7:**
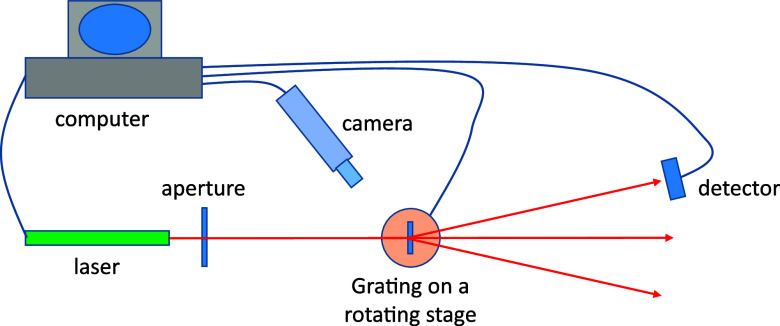
Setup for the measurement of the diffraction efficiency of the ion microbeam implanted gratings.

Diffraction efficiency of the ion microbeam implanted optical gratings was measured by a setup shown in Figure 7.

Two lasers were used for the diffraction efficiency measurements. The first one was a TECBL-10GC-405 semiconductor laser from World Star Technologies, working at a wavelength of 405 nm. The second laser was a SM600 Fabry–Perot pigtail semiconductor laser from Thorlabs, working at 640 nm, and connected to a fiber optics collimator to obtain a free space beam.

Lateral dimensions of the ion beam implanted gratings were generally 500 × 500 or 1 × 1 mm. Gratings were illuminated from the substrate side through a 500 μm diameter aperture to ensure that no laser light bypassed the gratings. Since the aperture was placed close to the sample, widening of the illuminating beam was negligible. Since grating constants were 30 μm, gratings consisted of 17 or 34 periods. However, amplitude fluctuations in the surface relief were relatively low so that the gratings could be considered homogeneous, in spite of the low number of periods.

Samples containing optical gratings were placed on a motorized rotation stage. A digital camera was used to align the illuminating laser beam on the gratings.

Diffraction efficiencies from diffraction orders of −4 to 4 were measured by using a Thorlabs PM100 detector, calibrated at the wavelengths of both lasers used. Averages of the diffraction efficiencies measured in the corresponding negative and positive orders were calculated.

Samples were always illuminated from the backside of the substrate so that the diffracted waves did not propagate inside the substrate. Corrections for the absorption of the sample and Fresnel reflections at the air-glass interfaces were taken into account. Thus, the reported diffraction efficiencies were net ones. Polarization of the illuminating beam was always perpendicular to the plane of incidence. Relative error of the diffraction efficiency was 5%.

Gross diffraction efficiencies were also measured. It was found that the implantations did not increase the absorption of the glass samples at visible wavelengths. Based on the measured amplitudes of the surface relief gratings and the diffraction efficiencies, and on the comparison of the implantation parameters of the actual experiment to the previous ones, where implantation through mask was performed,^33^ it was concluded that the strength of the implanted index-of-refraction gratings was negligible compared to that of the surface relief one.

Diffraction efficiency measurements were repeated with the incoming laser beam polarized parallel to the plane of incidence. The measurements gave the same results as those performed with the incoming laser beam polarized perpendicular to the plane of incidence.

Diffraction efficiencies of the same GG grating are the following: first-order diffraction efficiency was 25.5%, while that of the second order was 4.4%.

**Figure 8:**
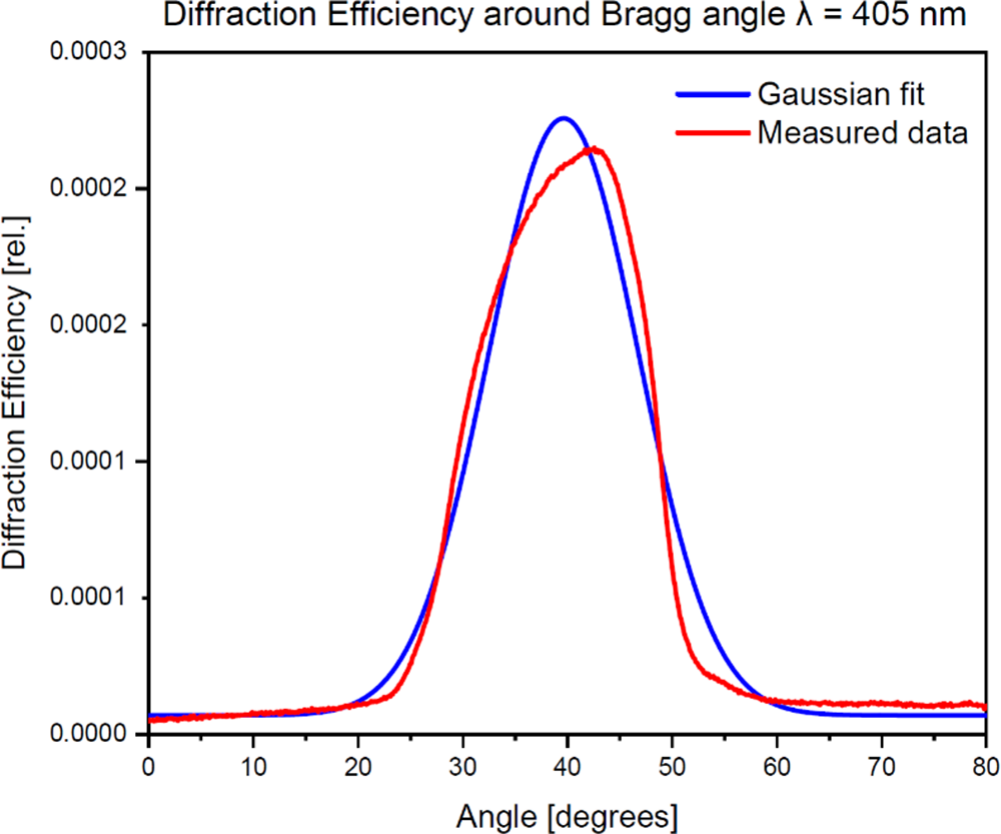
Angular selectivity of grating GG (IOG glass) vs the angle of incidence.

Dependence of the first-order diffraction efficiency of the GG grating as a function of the angle of incidence of the illuminating beam is shown in Figure 8. fwhm of the peak was 16.8°. The angle of 24° corresponds to the calculated Bragg angle. The lack of a pronounced Bragg effect indicates that the grating could be considered a thin one. The relative errors of the diffraction efficiency vs angle of incidence measurements were about 5%. The diffraction efficiency was measured at several hundred angles.

All other diffraction efficiency measurements were carried out at λ = 640 nm. Measured diffraction efficiencies, up to the fourth order, of the ion microbeam implanted optical gratings are summarized in Table 2.

**Table 2:** Measured and Calculated Diffraction Efficiencies of the Ion Microbeam Implanted Optical Gratings

name of the grating	sample material	implanted ion and charge	energy of the implanted ion [MeV]	implanted fluence [ion/cm^2^]	fitted amplitude [nm]	measured first-order diffraction efficiency	calculated first-order diffraction efficiency	measured second-order diffraction efficiency	measured third-order diffraction efficiency	measured fourth-order diffraction efficiency
GG (λ = 405 nm)	IOG glass	N^3+^	5	8 × 10^14^	211	0.26	0.34	0.044	0.0036	n.m.
GG (λ = 640 nm)	IOG glass	N^3+^	5	8 × 10^14^	211	0.20	0.22	0.014	0.0071	0.00078
GF2 (λ = 640 nm)	Pyrex glass	O^4+^	6	8.4 × 10^15^	265	0.26	0.28	0.047	0.0060	0.0014
GF4 (λ = 640 nm)	Pyrex glass	N^3+^	5	8.4 × 10^15^	193	0.18	0.18	0.016	0.0016	0.00084

It can be seen that first-order diffraction efficiency of grating GG at λ = 405 nm was 25% and that at 640 nm was 20%.

As for the implanted gratings in Pyrex samples, grating GF2 had the higher first-order diffraction efficiency of 26.2%. First-order diffraction efficiency of grating GF4 was 17.9%. The ratio of the first-order diffraction efficiency to the second-order one at 640 nm is the following: 14 for GG, 5.5 for GF2 and 11.3 for GF4. This fact proves that the gratings are quasi-sinusoidal.

Diffraction efficiency of thin gratings is given by the well-known Raman-Nath equation^57,58^:
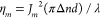
1where η_*m*_ is diffraction efficiency in the *m*-th order, *J_m_* is the m-order Bessel function, Δ*n* is the maximum difference of refractive index in the grating, *d* is the peak-to-peak amplitude of the grating, and λ is the wavelength. In the case of surface relief gratings, Δ*n* = *n*_grating_ – *n*_air_.

First-order diffraction efficiencies of a part of the ion microbeam implanted gratings were calculated using the Raman-Nath equation and compared to the measured first-order diffraction efficiencies. The results are presented in Table 2.

It can be seen in Table 2 that the measured first-order diffraction efficiencies are slightly lower than the calculated ones. The differences between calculated and measured diffraction efficiencies, measured at λ = 640 nm, are 9, 7, and 3% of the calculated ones. This can be attributed to the fact that eq 1 is valid for perfectly sinusoidal gratings, while the profiles of the gratings studied here had some higher harmonics. As shown in Table 2, each grating diffracted a small part of the incoming laser light into higher orders.

### 2.7 Discussion

The proposed and demonstrated method produced efficient surface relief diffraction gratings of a quasi-sinusoidal profile via a corresponding sinusoidal subperiod modulation of the implanted fluence.

The evident drawback of the method is that a Tandetron accelerator with a microbeam facility has to be used for the production of the gratings. Photonic foundries and femtosecond laser writing facilities are much more common and are more easily accessible.

However, the proposed method has some advantages over both microelectronic technology and femtosecond laser writing. It is a single-step process, i.e., the implanted gratings are ready for use. The only optional postprocessing could be a thermal annealing, if the implantation would produce considerable absorption increase (especially in optical crystals). As it was mentioned earlier, fabrication of gratings and other integrated optical elements by microelectronic technology involves several steps. As for femtosecond laser writing, recent articles report on the use of two-step laser irradiation to achieve some specific goals.

Implantation with microbeams, a so-called swift heavy ion (SHI), offers the possibility of a high degree of control of the properties of the integrated optical elements to be fabricated. For example, in the case of optical gratings, by varying the energy, the implanted fluence, and the current density (flux) of the microbeam, one could produce all kinds of mixed gratings. Certain values of those parameters would produce three gratings: one of index of refraction, another of absorption, and one-third of surface relief. In the present work, thanks to the very high current density in the microbeam, only surface relief gratings were produced. As for the production of index-of-refraction gratings, by only changing the energy of the microbeam, one could write buried gratings or other structures at various depths of the sample, i.e., 3D structures could also be produced.

Another advantage of the proposed method over femtosecond laser writing is its linearity. Femtosecond laser writing with very high resolution relies on nonlinear absorption.

Wang et al. deal with the surface relief gratings in their recent review.^59^ They presented both historical and state-of-the-art achievements in that field. Their review focused on the research status of metal-film reflection gratings and multilayer dielectric-film transmission and the reflection gratings. Their analysis showed that metal-film gratings were not able to achieve high diffraction efficiency and antilaser-damage threshold values due to the inherent absorption characteristics of materials. Although the grating produced in the present work cannot be compared to those two classes of surface relief gratings, it can be expected that the ion microbeam implantation technique could result in higher amplitude of the surface relief gratings and hence in higher diffraction efficiency. Antilaser-damage of the implanted grating threshold basically depends on that of the target material.

## 3 Conclusions

A method was devised and realized for the fabrication of surface relief optical gratings of quasi-sinusoidal profile in optical glasses using microbeams of medium-mass ions in the 5–6 MeV energy range. The method is based on the appropriate modulation of the implanted fluence across the grating lines. Target materials were IOG and Pyrex glasses. Microbeams of nitrogen and oxygen ions were used for fabrication of the gratings.

Sine functions were fitted to the measured profiles of the surface relief gratings. The measured highest amplitude of an ion beam implanted grating was 265 nm.

The proposed method has some advantages over the existing fabrication methods: It is a single-step process. It is linear, i.e., the amplitude of the surface relief gratings was proportional to the implanted fluence. Besides surface relief gratings, other types of gratings could also be fabricated using the proposed method by carefully controlling energy, current density, and implanted fluence. Fabrication of three-dimensional structures containing buried index-of-refraction gratings at various depths could also be feasible.

According to our experiences with ion microbeam implanted channel waveguides, using ion microbeams of higher energy, above 10 MeV, could considerably reduce the necessary implanted fluence.^60^

Such optical gratings can be used in biochemical sensors for coupling light into and out of waveguides.^61^ Such grating couplers can work in the visible and near-infrared wavelength range. Other elements of biochemical sensors, e.g., planar and channel waveguide, can also be fabricated by ion beam techniques.^15^
